# Investigation of Stilbenoids as Potential Therapeutic Agents for Rotavirus Gastroenteritis

**DOI:** 10.1155/2015/293524

**Published:** 2015-08-26

**Authors:** Judith M. Ball, Fabricio Medina-Bolivar, Katelyn Defrates, Emily Hambleton, Megan E. Hurlburt, Lingling Fang, Tianhong Yang, Luis Nopo-Olazabal, Richard L. Atwill, Pooja Ghai, Rebecca D. Parr

**Affiliations:** ^1^Department of Pathobiology, Texas A&M University, College Station, TX 77843, USA; ^2^Department of Biological Sciences & Arkansas Biosciences Institute, Arkansas State University, Jonesboro, AR 72401, USA; ^3^Department of Biology, Stephen F. Austin State University, Nacogdoches, TX 75962, USA

## Abstract

Rotavirus (RV) infections cause severe diarrhea in infants and young children worldwide. Vaccines are available but cost prohibitive for many countries and only reduce severe symptoms. Vaccinated infants continue to shed infectious particles, and studies show decreased efficacy of the RV vaccines in tropical and subtropical countries where they are needed most. Continuing surveillance for new RV strains, assessment of vaccine efficacy, and development of cost effective antiviral drugs remain an important aspect of RV studies. This study was to determine the efficacy of antioxidant and anti-inflammatory stilbenoids to inhibit RV replication. Peanut (*A. hypogaea*) hairy root cultures were induced to produce stilbenoids, which were purified by high performance countercurrent chromatography (HPCCC) and analyzed by HPLC. HT29.f8 cells were infected with RV in the presence stilbenoids. Cell viability counts showed no cytotoxic effects on HT29.f8 cells. Viral infectivity titers were calculated and comparatively assessed to determine the effects of stilbenoid treatments. Two stilbenoids, trans-arachidin-1 and trans-arachidin-3, show a significant decrease in RV infectivity titers. Western blot analyses performed on the infected cell lysates complemented the infectivity titrations and indicated a significant decrease in viral replication. These studies show the therapeutic potential of the stilbenoids against RV replication.

## 1. Introduction

The mechanisms of RV-induced diarrhea are multifactorial and include both secretory and malabsorptive diarrhea components. Despite much effort, we do not have a complete understanding of RV pathophysiology [1]. Vaccine strategies against RV-associated diarrhea aim at stimulating the immune system using either attenuated live RV or RV proteins [2]. There are two licensed RV vaccines in the United States, RotaTeq, produced by Merck, and Rotarix, produced by GlaxoSmithKline. Both are effective in preventing severe diarrhea in vaccinated children [3, 4]. Recently, a new RV vaccine, Rotavac, was developed using a strain of the RV that was isolated, manufactured, and tested in India by Hyderabad-based Bharat Biotech International, Ltd. [5]. These vaccines are designed to protect against common RV strains and therefore are dependent on the genetic stability of the viruses. Reassortment events are common and may lead to new virulent RV strains that may not be averted by the current vaccines [6]. Likewise, the zoonotic nature of RV infections supports the argument to continue to survey for emerging RV strains arising from interspecies transmission with potential of vaccine failures [7]. The licensed RV vaccines mentioned above are less efficacious in countries of sub-Saharan Africa and SE Asia where they are needed most [8–11]. Furthermore, high costs, limited availability, and poor logistics for the distribution of the vaccines are challenging problems for the developing world [3]. Consequently, the development of cost effective, easily distributed, novel, and host-oriented antiviral paradigms is needed that affect a wide range of RV strains and reduce the disease burden of RV infections. Taking advantage of the antioxidant and anti-inflammatory properties of a natural product to treat RV infections meets the principles of a novel therapeutic strategy that has an antiviral effect.

Stilbenoids are phenolic compounds derived from the phenylpropanoid/acetate pathway. Among these compounds,* trans*-resveratrol (t-Res) is the most extensively studied stilbenoid which demonstrates strong antioxidant and chemopreventive properties [12]. Additionally, studies examining resveratrol and its derivatives have demonstrated antiviral properties. Resveratrol strongly inhibits the replication of influenza virus in MDCK cells and improves survival and decreased pulmonary viral infectivity titers in influenza virus-infected mice. Furthermore, resveratrol exhibited no toxic effects* in vitro* or* in vivo* [13]. However, another study tested the effects of 20 *μ*M and 40 *μ*M concentrations of resveratrol incubated for 24 and 48 hours postinfection with polyomavirus in 3T3 and HL60 cells. The results showed cytotoxicity in a time- and dose-dependent manner and inhibition of polyomavirus DNA synthesis. There was no cytotoxic effect to either cell line with 0.02% dimethyl sulfoxide (DMSO) alone [14]. Another study identified resveratrol derivatives with potent anti-HSV-1 and HSV-2 activity. Several trimeric and tetrameric derivatives showed antiherpetic activity at single-digit micromolar concentrations [15].

**Figure 1:**
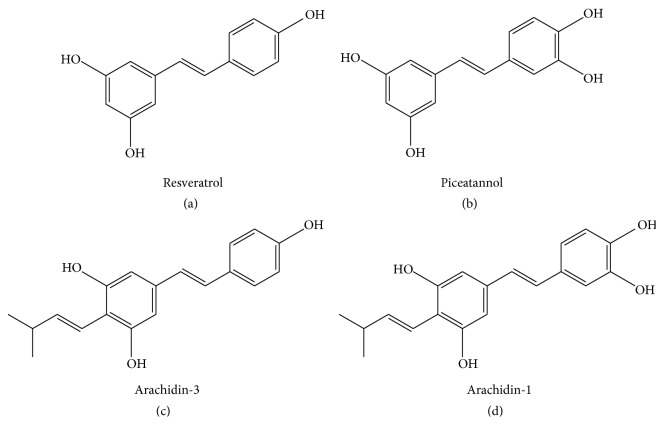
Chemical structures of the four stilbenoids tested. All compounds are shown in trans-isomers. (a) Resveratrol (t-Res). (b) Piceatannol (t-Pa). (c) Arachidin-3 (t-A3). (d) Arachidin-1 (t-A1).

Stilbenoids are produced by a group of plants which includes grapes, peanuts, and some berries [12, 16, 17].* trans*-Piceatannol (t-PA) is a hydroxylated analog of resveratrol found in grapes and in minor quantities in peanuts.* trans*-Arachidin-1 (t-A1) is a prenylated (3-methyl-1-butenyl) analog of piceatannol, whereas* trans*-arachidin-3 (t-A3) is a prenylated (3-methyl-1-butenyl) analog of resveratrol (Figures 1(a)–1(d)). Both t-A1 and t-A3 are produced in peanuts upon fungal challenge. These stilbenoids can be extracted from some plants but are not suitable for many applications in the food/pharmaceutical sectors due to the overall low concentration of stilbenoids in the plant extracts. To deliver a highly defined and stilbenoid-enriched product, hairy root cultures of peanut (*A*.* hypogaea*) have been established in a bioproduction system that produces increased levels of stilbenoids, including t-A1 and t-A3, upon treatment with elicitors [18, 19]. t-PA and t-Res are commercially available, but t-A1 and t-A3 are still in an experimental stage resulting in an opportunity to explore new antiviral biological activity.

This study assessed the therapeutic potential of four stilbenoids, t-Res, t-PA, t-A1, and t-A3 (Figures 1(a)–1(d)), to inhibit RV infections in culture using a cloned human intestinal cell line, HT29.f8 [20]. The hypothesis for this work is that stilbenoids will modulate the viral load of RV generated during an infection. Two sets of experiments were performed in which different concentrations of the stilbenoids and two time points postinfection were evaluated. To determine the effect of the stilbenoids on the amount of virus produced during an infection, viral infectivity titers were determined using the supernatants for each of the different treatments (10 *μ*M and 20 *μ*M stilbenoids) collected at 12 and 24 hours postinfection (hpi). The viral infectivity titers produced in cells treated with the stilbenoids using a focus forming unit (FFU) assay were compared to the virus infectivity titers generated from RV infections alone and RV infections with 0.02% DMSO. The results were reported as infectious virus particles/mL. Western blot analyses using the cell lysates generated from these experiments demonstrated the presence of the nonstructural RV protein (NSP4 nonstructural protein 4), a multifunctional viral protein that is essential for virus replication and the production of infectious virus particles [21].

## 2. Materials and Methods

### 2.1. Cells, Virus, and Reagents

**Figure 2:**
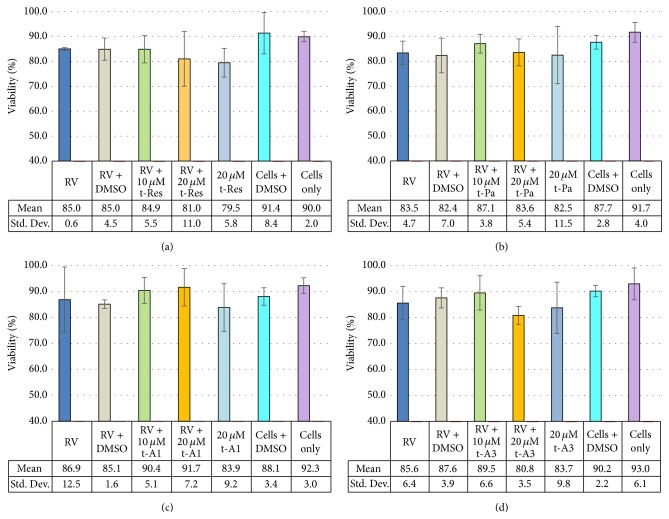
HT29.f8 Cell viability at 24 hpi with stilbenoids. (a) Resveratrol (t-Res). (b) Piceatannol (t-Pa). (c) Arachidin-1 (t-A1). (d) Arachidin-3 (t-A3).

The objective of the study was to test the effect(s) of the four stilbenoids on RV replication in HT29.F8 cells with variable concentrations of the stilbenoids and different collection times. The dose was based on a previous study that assessed the effects of 20 *μ*M resveratrol and 0.02% DMSO on two cell lines infected with polyomaviruses [13, 14]. Another study utilized different concentrations of DMSO and higher concentrations of resveratrol (50, 100, and 200 *μ*M) on influenza A-infected cell line. Fifty and 100 *μ*M concentrations of resveratrol were not cytotoxic to the cells [13, 14]. Based on these results, we choose to test 10 *μ*M and 20 *μ*M concentrations of each stilbenoid solubilized in DMEM with 0.02% DMSO. A total of five experimental sets were performed per stilbenoid. In the first experimental set, cells were infected with SA114F RV at a multiplicity of infection (MOI) of 2 as previously reported [22]. In the second experimental set, 0.02% DMSO was added to the RV infection to prove that 0.02% DMSO used to solubilize the stilbenoids had no effect on cell viability or production of RV. In the third and fourth experimental sets, 10 *μ*M or 20 *μ*M concentrations of the stilbenoids, respectively, were solubilized in 0.02% DMSO in DMEM, added to the RV inoculum, and used to infect the cells. The fifth set was uninfected HT29.f8 cells treated with the stilbenoids. The sixth set was uninfected HT29.f8 cells treated with 0.02% DMSO, and the seventh set was uninfected HT29.f8 cells alone (Figure 2). Each experimental set was tested in four wells of a 24-well tissue culture (TC) plate. The media from the four wells were pooled and centrifuged and the supernatants were stored at −80°C and used to determine viral infectivity titers. The cells were collected in PBS, frozen, thawed 3 times, and centrifuged. The supernatants were collected as the cell lysates and stored at −80°C until used in western blot assays. Viral infectivity titers were performed in triplicate using two assays, the focus forming units (FFU) and the plaque forming units (PFU) assays. Equal amounts of the cell lysates were used in western blot assays to resolve the viral proteins and probe for RV NSP4.

### 2.2. Bioproduction and Purification of the Stilbenoids

Hairy roots of peanut cv. Hull (line 3) were cultured in at least twenty 250 mL flasks, each containing 50 mL of MSV medium as previously described [18, 23]. At day nine of the hairy root culture, the spent medium from each flask was removed and replaced with elicitation medium (fresh MSV medium with 9 g/L methyl-*β*-cyclodextrin (Cavasol W7 M)) and incubated in the dark at 28°C for an additional 72 h to induce synthesis and secretion of stilbenoids into the culture medium as recently described [19]. After the elicitation period, the culture medium was removed from each flask and combined. This pooled medium was mixed with an equal volume of ethyl acetate in a separatory funnel to extract the stilbenoids as described before [18]. The ethyl acetate phase was recovered and was dried in a Rotavapor (Buchi), and t-A1 and t-A3 were purified from the extract by HPCCC as follows. The dried ethyl acetate extract was resuspended in HPCCC solvent system (hexane : ethyl acetate : methanol : water (4 : 5 : 3 : 3)) and injected into a Spectrum (Dynamic Extractions) HPCCC system. The upper phase of the solvent system was used as the stationary phase and the chromatography was monitored at UV 340 nm. Fractions were collected every 30 s, dried in a SpeedVac, and analyzed by HPLC.

HPLC analyses were performed in a Dionex Summit system, equipped with a photodiode array (PDA) detector. The separation was performed on a SunFire C_18_, 5 *μ*m, 4.6 × 250 mm column (Waters) at 40°C at a flow rate of 1.0 mL/min. The mobile phase consisted of 2% formic acid in water (A) and methanol (B). The method started with 100% A for 1 min. Then a linear gradient was performed from 40% A and 60% B to 35% A and 65% B (1 to 20 min), followed by a linear gradient from 35% A and 65% B to 100% B (20 to 25 min). Then the column was washed with 100% A for 5 min (25 to 30 min). Elicited peanut seed-derived t-A1 and t-A3 were used as reference standards [23].

Purity of the fractions obtained after HPCCC was monitored by HPLC using UV absorbance at 280, 320, and 340 nm. Selected fractions also were checked for purity by mass spectrometry using an UltiMate 3000 ultrahigh performance liquid chromatography (UHPLC) system (Dionex, Thermo Scientific) coupled with a LTQ XL linear ion trap mass spectrometer (Thermo Scientific) as described in Marsh et al. [24]. HPCCC fractions containing t-A1 and t-A3 with over 95% purity based on HPLC analysis (UV 340 nm) were combined, dried under a nitrogen stream, and used for viral assays. The dry mass of the purified stilbenoids was reconstituted in 0.02% DMSO with 1 *μ*g/m trypsin (Worthington Biochemical, Lakewood, NJ) in DMEM medium. To compare the results between nonprenylated stilbenoids (t-Res and t-PA) and their prenylated analogs (t-A3 and t-A1, resp.) the synthetic/commercially available t-Res (Sigma-Aldrich) and t-PA (Alexis) were used in this study.

### 2.3. Cell Lines and Virus

MA104 cells were obtained from ATCC (Rockville, MD) and the HT29.F8 cells, a spontaneously polarizing cell line, were derived from the parent human adenocarcinoma (HT29) intestinal line [20]. The cell lines were confirmed to be free of mycoplasma contamination using the MycoFind mycoplasma PCR kit version 2.0 (Clongen Laboratories, LLC). RV SA11 clone 4F (P[1] and G[3] genotype) [25] was grown and titered in MA104 cells and stored at −80°C. Stilbenoid efficacy against RV was tested using HT29.f8 cells.

### 2.4. Viability Assay

The percentage of live/dead cells was calculated using the trypan blue dye exclusion assay as previously outlined [26]. Briefly, a cell suspension of ~10^6^ cells/mL was diluted 1 : 1 with a 0.4% trypan blue solution and loaded onto a hemocytometer. The number of stained cells and total number of cells were counted, and the calculated percentage of unstained cells was reported as the percentage of viable cells. To determine if the 0.02% DMSO that was used to solubilize the hydrophobic stilbenoids adversely affected the life span of HT29.F8 cells, viability assays were performed with RV alone, RV with 0.02% DMSO, cells with 0.02% DMSO, cells alone, and RV with 0.02% DMSO with 10 *μ*M and 20 *μ*M stilbenoids using the trypan blue cell exclusion assay as described [26].

### 2.5. Virus Quantification

To test the biological activity of the stilbenoids on RV infections, both FFU and PFU assays were performed as previously described [27, 28]. MA104 cells were grown to 80% confluence in 24-well tissue culture plates (Corning Life Sciences), starved for fetal bovine sera 12 h prior to infection, and then infected with RV SA114F. Briefly, the SA114F RV stock was sonicated (5 min using a cup horn attachment and ice bath in a Misonix Sonicator 3000, Misonix, Inc., Farmingdale, NY) and incubated in serum-free DMEM with 1 *μ*g/mL trypsin (Worthington Biochemical, Lakewood, NJ) for 30 min at 37°C. The activated viral inoculum was incubated with the cells for 1 h at 37°C in 5% CO_2_ at an MOI of 2. The inoculum was replaced with serum-free DMEM supplemented with 1 *μ*g/mL trypsin and incubated for 12 and 24 hpi. The supernatants were collected, clarified at 300 ×g for 5 min, and stored at −80°C. The cells were washed in cold Dulbecco's PBS, 1X (Caisson Laboratories, Smithfield, UT), and released from the plates using a 0.25% trypsin-EDTA solution (1X) (Caisson Laboratories, Smithfield, UT). After the addition of DMEM with 5% FBS, the cells were resuspended in cold PBS and dilutions were prepared for live/dead cell counts (see Section 2.4). The balance of the cells was used to prepare cell lysates by subjecting them to repeated freeze-thaws three times, clarified at 300 ×g for 10 min. Media (supernatant) were collected, clarified at 300 ×g for 10 min, and stored at −80°C. Both the cell lysates and supernatants were stored at −80°C. Viral infectivity titers were done in triplicate by indirect immunofluorescent staining of MA104 monolayers infected with serial dilutions of the supernatants. The average number of fluorescent foci was calculated for three wells and used to determine the number of focus forming units/mL (FFU/mL) [29]. Since the RV viral infectivity titers are critical to our conclusion, two assays were used. The FFU data showed no difference in viral infectivity titers at 12 hpi using 10 *μ*M concentrations (data not shown). Therefore, we chose to perform plaque forming unit (PFU) assays comparing the two controls, RV alone and RV with 0.02% DMSO to 20 *μ*M of the stilbenoids at 24 hpi. Plaque forming assays were performed in triplicate as outlined above for the FFU assays, except after the 1-hour infection; the virus inoculum was replaced with 3 mL of a medium overlay (1 : 1 mixture of 1.2% agarose (Apex Low Melting Point Agarose, Genesee Scientific Inc.) and complete 2 × MEM containing 0.5 *μ*g/mL trypsin) and incubated at 37°C in 5% CO_2_ for 3 to 4 days or until plaques became visible. A neutral red overlay (1 : 1 mixture of 1.2% agarose with an equal volume of serum-free 2 × MEM containing 50 *μ*g/mL neutral red) was prepared and 2 mL per well of stain overlay was added on top of the first agarose/medium overlay. The six-well plates were incubated at 37°C until plaques were visible (approximately 4 to 24 h). The individual plaques were counted, and the titers were calculated as follows: number of plaques  ×  1/dilution factor × 1/(mL of inoculum) = PFU/mL.

### 2.6. Statistical Analysis

Data are expressed as mean ± SD, and comparisons were statistically evaluated by analysis of variance (ANOVA) and Student's *t*-tests using Excel (significance level, *p* ≤ 0.05).

### 2.7. Protein Quantification and Western Blot Assays

The micro bicinchoninic acid (BCA) protein assay was employed to quantify protein concentrations using bovine serum albumin as the standard per manufacturer's protocol (Thermo Scientific Pierce). One microgram of total protein from each sample was separated by 12.5% SDS-PAGE, electroblotted onto nitrocellulose membranes, and probed with NSP4 peptide-specific antibodies [30, 31] and reactive bands were visualized by the addition of HRP-conjugated IgG and Super Signal West Pico chemiluminescent substrate (Pierce) followed by exposure to Kodak X-OMAT film [22, 32, 33].

## 3. Results and Discussion

### 3.1. Bioproduction of Stilbenoids in Hairy Root Cultures of Peanut

**Figure 3:**
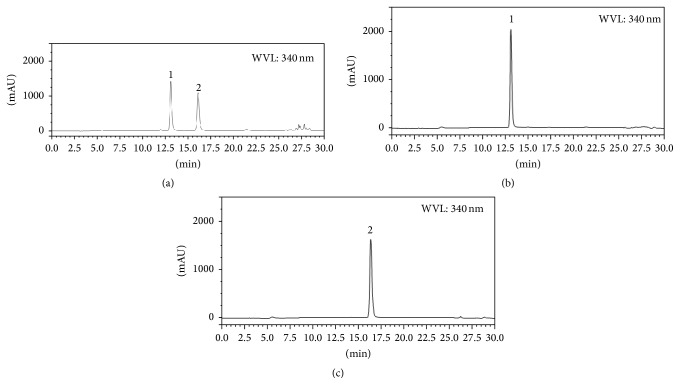
HPLC analysis of stilbenoids. The *x*-axis is time in minutes, and the *y*-axis is the absorbance at 340 nm. (a) HPLC chromatogram of ethyl acetate extract of the medium of hairy root culture of peanut treated with methyl-*β*-cyclodextrin for 72 h. Compounds: (1) arachidin-1 and (2) arachidin-3. (b) HPLC chromatogram of (1) arachidin-1 purified by HPCCC. (c) HPLC chromatogram of (2) arachidin-3 purified by HPCCC.

To produce the stilbenoids t-A1 and t-A3 we used our previously established hairy root line 3 from peanut cv. Hull. These hairy roots are capable of synthesizing and secreting t-Res, t-A1, and t-A3 into the culture medium upon treatment with the elicitor sodium acetate [18]. Depending on the period of elicitor treatment, the levels and types of stilbenoids found in the medium can be modified [18]. To study the effect of other elicitors on production of t-A1 and t-A3 we tested different elicitors, including methyl-*β*-cyclodextrin (CD). In preliminary experiments different doses of CD were added to the hairy root cultures for different periods between 0 and 96 h (data not shown). A 72 h treatment of 9 g/L CD was selected based on production of the highest levels of t-A1. As shown in Figures 3(a)–3(c), t-A1 and t-A3 were the major stilbenoids present in the culture medium. t-Res was present in very small amounts in these extracts. To purify t-A1 and t-A3, ethyl acetate extracts were made from the culture medium and subjected to HPCCC (high performance countercurrent chromatography). The solvent system was adapted from a previously used CPC (centrifugal partition chromatography) system which was effective in purifying t-A1 and t-A3 from hairy root culture medium extracts [16]. The only modifications were the replacement of heptane for hexane and ethanol for methanol. The separation was effective and comparable to the one achieved before [16]. Thus high yields of highly purified fractions of t-A1 and t-A3 were achieved and were used in the antiviral assays.

### 3.2. Viability of HT29.F8 Cells in the Presence of 0.02% DMSO

The percentage of live/dead cells was calculated using the trypan blue exclusion dye assay (Figures 2(a)–2(d)). At 24 hpi, the cell viability between all groups tested (HT29.f8 cells with RV, HT29.f8 cells with RV and 0.02% DMSO, HT29.f8 cells with RV and 10 *μ*M stilbenoids, HT29.f8 cells with RV and 20 *μ*M stilbenoids, HT29.f8 cells with 20 *μ*M stilbenoids only, HT29.f8 cells with 0.02% DMSO, and HT29.f8 cells only) was not statistically significantly different (*p* < 0.05). These data revealed that the addition of RV increases cell death, but not significantly in the time frame examined. Also, the addition of 20 *μ*M concentrations of the stilbenoids decreased cell viability but not significantly, while the addition of 0.02% DMSO to the culture system did not adversely affect the viability of the HT29.f8 cells in culture or diminish viral replication (Figures 2(a)–2(d) and 6). These data demonstrate that HT29.f8 cells were not adversely affected by RV, 0.02% DMSO, or concentrations up to 20 *μ*M of the four stilbenoids tested (t-Res, t-PA, t-A1, or t-A3).

### 3.3. The Effects of Stilbenoids on the Production of Infectious Rotavirus Particles

**Figure 4:**
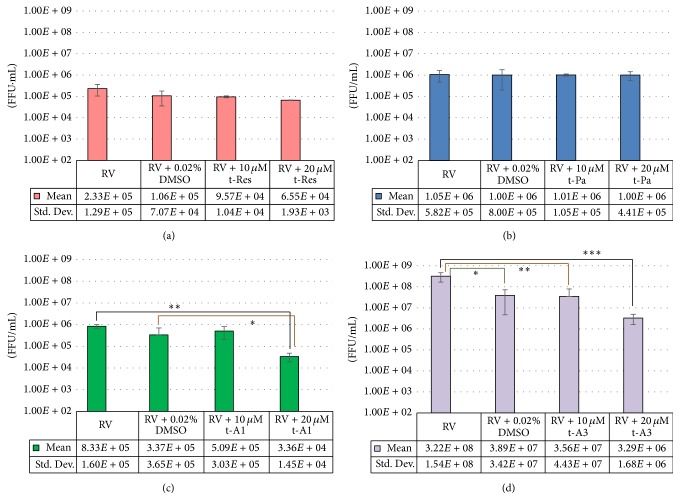
Quantification of progeny RV via focus forming units/mL (FFU/mL) at 24 hours postinfection. HT29.8 cells were infected with RV, with RV containing 0.02% DMSO, or 10 *μ*M/20 *μ*M of (a) resveratrol (t-Res). (b) Piceatannol (t-Pa). (c) Arachidin-1 (t-A1) ^*∗*^ *p* = 0.02 and ^*∗∗*^ *p* = 0.001 and (d) arachidin-3 (t-A3) ^*∗*^ *p* = 0.04, ^*∗∗*^ *p* = 0.04, and ^*∗∗∗*^ *p* = 0.02.

Viral infectivity titers were determined using FFU assays from the supernatants of RV-infected HT29.f8 cells treated with stilbenoids (10 *μ*M and 20 *μ*M t-Res, t-PA, t-A1, or t-A3). Supernatants collected at 12 hpi were equivalent to the RV-infected control cells (data not shown). Similarly, at 24 hpi, the 20 *μ*M concentrations of the nonprenylated stilbenoids, t-Res and t-PA both, demonstrated no change in the virus titer when compared to the RV-infected control (Figures 4(a) and 4(b)). However at 24 hpi, the 10 *μ*M concentrations of t-A1 generated a tenfold decrease in virus infectivity titer when compared to the RV-infected control supernatants (*p* = 0.02), and the 20 *μ*M concentrations of t-A1 generated a twenty-fivefold decrease in virus infectivity titer when compared to the RV-infected control supernatants (*p* = 0.001) (Figure 4(c)), However, there was a statistical difference between RV and RV with DMSO with an eightfold decrease in virus infectivity titers with RV and DMSO (*p* = 0.04) (Figure 4(d)). The 10 *μ*M concentrations of t-A3 generated a ninefold decrease in virus titer when compared to the RV-infected control supernatants (*p* = 0.04), and the 20 *μ*M concentrations of t-A3 generated a ninety-eightfold decrease in virus titer when compared to the RV-infected control supernatants (*p* = 0.02) (Figure 4(d)).

**Figure 5:**
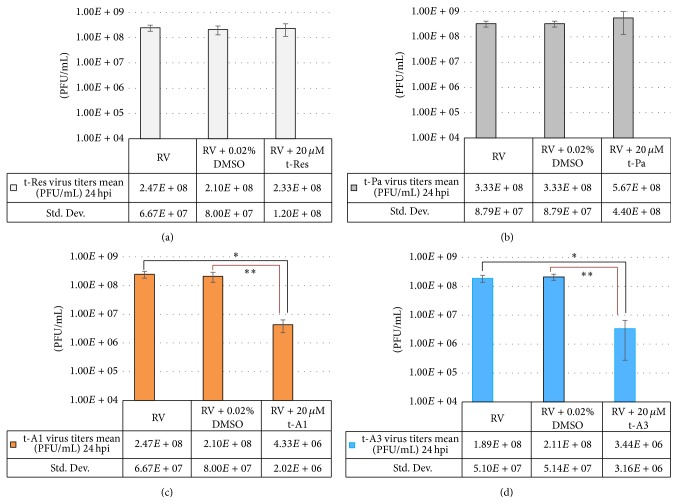
Quantification of progeny RV in plaque forming units/mL (PFU/mL) at 24 hpi. HT29.8 cells were infected with RV, with RV containing 0.02% DMSO, or 20 *μ*M of (a) resveratrol (t-Res). (b) Piceatannol (t-Pa). (c) Arachidin-1 (t-A1). ^*∗*^Statistically significant *p* = 0.02. ^*∗∗*^Statistically significant *p* = 0.04. (d) Arachidin-3 (t-A3). ^*∗*^Statistically significant *p* = 0.02. ^*∗∗*^Statistically significant *p* = 0.02.

Since the data generated with the FFU assays were critical to test our hypothesis, plaque forming unit assays (PFU assays) were performed to corroborate the results obtained from the FFU assays. The PFU assays were performed using the same supernatants that were utilized for the FFU assays. Plaques were counted and the average of three experiments was calculated and graphed as PFU/mL (Figures 5(a)–5(d)).

The data produced using the PFU assays showed similar fold differences as shown with the FFU assays (Figures 5(a)–5(d) and 4(a)–4(d), resp.). Using the ANOVA and Student's *t*-test, the average and standard deviations were calculated and graphed (Figures 4(a)–4(d) and 5(a)–5(d)). The PFU experiments using t-Pa and t-Res showed no statistical differences between the controls, RV only and RV with DMSO, RV with 10 *μ*M t-Pa/t-Res, or RV with 20 *μ*M t-Pa/t-Res (Figures 5(a) and 5(b)). However, the experimental data from t-A1 PFU assays demonstrated a fifty-sevenfold difference from the control, RV only, and a forty-ninefold difference from the control, RV with DMSO that was statistically significant (*p* = 0.02 and 0.04, resp.) (Figure 5(c)). Likewise, the experimental data from 20 *μ*M t-A3 PFU assays demonstrated a fifty-fivefold difference from the control, RV only, and a sixty-onefold difference from the control, RV with DMSO that was statistically significant (*p* = 0.02 and 0.02, resp.) (Figure 5(d)). Both assays show a significant decrease in RV infectivity titers in the presence of 20 *μ*M t-A3.

### 3.4. Western Blot (WB) Analyses Imply Differences in RV Replication

**Figure 6:**
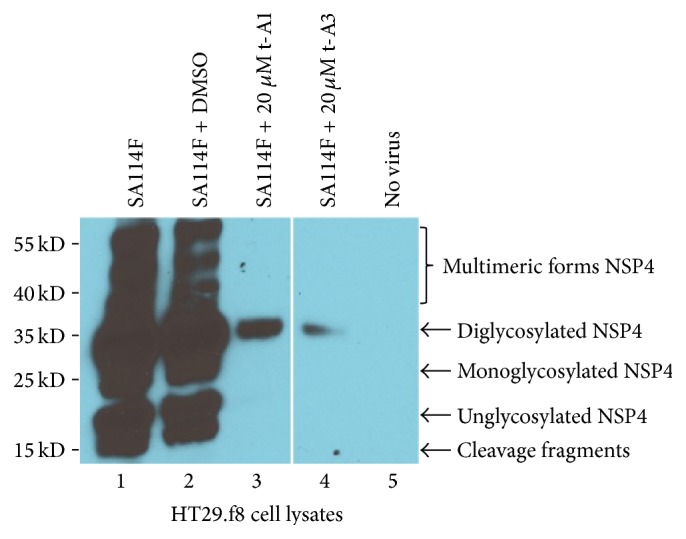
Western blot analysis of HT29.f8 cell lysates. Five micrograms of HT29.f8 cell lysates was separated on a 12.5% SDS-PAGE, electroblotted onto nitrocellulose membranes, probed with rabbit anti-NSP4_150–175_ peptide-specific and goat anti-rabbit HRP-conjugated IgG, and visualized with Super Signal West Pico chemiluminescent substrate (Pierce) followed by exposure to Kodak X-OMAT film. (Lane 1) RV-infected HT29.f8 cells and (Lane 2) RV-infected HT29.f8 cells with 0.02% DMSO, respectively, show cleavage fragments and unglycosylated, mono-, diglycosylated, and multimeric forms of NSP4. (Lane 3) RV-infected HT29.f8 cells with 20 *μ*M t-A1 and (Lane 4) RV-infected HT29.f8 cells with 20 *μ*M t-A3 only show the diglycosylated form of NSP4. (Lane 5) HT29.f8 with no virus showed NSP4 banding pattern.

To complement and visualize the differences demonstrated in the viral infectivity titers between RV alone and RV with DMSO, 20 *μ*M t-A1, and 20 *μ*M t-A3, western blot assays were performed as previously described [22, 32, 33]. Using equal amounts of protein of the corresponding cell lysates, the nonstructural viral protein 4, NSP4, was detected in all RV-infected cell lysates. The western blot data of the RV and RV with DMSO both demonstrated relatively equal amounts of multimeric forms and diglycosylated (fully glycosylated), monoglycosylated, and cleavage fragments of NSP4 and suggests that 0.02% DMSO does not affect the amount of NSP4 produced during a RV infection (Figure 6, Lanes 1 and 2). The presence of multimeric forms of NSP4 has been previously studied [34–36]. The results for RV with t-A1 and t-A3 display a relatively small amount of the fully glycosylated form of NSP4 (Figure 6, Lanes 3 and 4). This indicates viral replication is negatively affected by 20 *μ*M of both t-A1 and t-A3. Cell lysates without RV (Figure 6, Lane 5) reveal no bands and show the specificity of the anti-NSP4 antibodies.

## 4. Conclusions

Our data show a dose- and time-dependent decrease in viral progeny when RV and prenylated stilbenoids (t-A1 or t-A3) were incubated with the human intestinal cell line HT29.F8. The presence of the nonstructural viral protein NSP4 in the western blot assays confirms the RV infection and indicates the virus was replicating in the HT29.f8 cells. The prenylated stilbenoids, t-A1 and t-A3, significantly are more lipophilic than either of the nonprenylated t-Res or t-PA molecules. The prenylated side chain increases the lipophilicity of the molecules to which it is attached. Consequently, prenylation promotes association with and penetration through cell membranes. An increase in lipophilicity often correlates positively with increased biological activity within different groups of compounds of similar structure [37, 38]. Several delivery systems including emulsions and nanoparticles have been tested for the delivery of lipophilic, bioactive natural products [39]. Depending on the application, these delivery systems may be applicable to t-A1 and t-A3 and should be tested to advance their development as potential therapeutic agents.

Although the molecular mechanisms for the protective effect of t-A1 and t-A3 are not known, the inhibition of viral replication could be attributed to the antioxidative and anti-inflammatory properties of the constituent stilbenoids. In a previously published paper, t-A1 and t-A3 have been shown to modulate the cannabinoid receptors at micromolar levels [40]. The experimental data of this previous study show that t-A3 acts as a competitive cannabinoid receptor 1 (CB1R) antagonist, whereas t-A1 antagonizes CB1R agonists by both competitive and noncompetitive mechanisms [40]. It is interesting that the HT29 cell line, the parent cell line of HT29.f8, expresses cannabinoid receptors [41], and receptor expression should be investigated on the HT29.f8 cloned cells. These receptors are part of the endocannabinoid signaling system which is well known to regulate gastrointestinal functions, such as gastric emptying, secretion, and intestinal motility [42, 43]. Hence it is reasonable to propose a connection between the cannabinoid receptor functions and the mechanism of RV gastroenteritis. In a study on colorectal cancer, cannabinoid receptor (CB1 and CB2) agonists were shown to have an effect on apoptosis through a TNF*α*-mediated increase in ceramide production [44]. Another study on breast cancer shows the receptor agonists inhibit adenylyl cyclase activity, cAMP, and PKA activity resulting in the downregulation of gene transcription [45, 46]. A cAMP-dependent PKA mechanism also appears to be important in RV pathogenesis in a human intestinal cell line, Caco2 [47].

Recently, cannabinoid receptor antagonists were proposed as potential therapeutic agents against hepatitis C virus by modulating lipid homeostasis [48]. A study by Gaunt et al. [49] using RV-infected MA104 cells demonstrates a dose-dependent reduction in virus infectivity and viral RNA production with the addition of TOFA (5-(tetradecyloxy)-2-furoic acid), an inhibitor of the fatty acid synthase enzyme complex [49]. Further, the infectivity of RV in ACC1 knockdown cells was reduced by 8.5-fold (significant, *p* = 0.01) with siRNA directed against ACC1, the gene that encodes the enzyme catalyzing the rate-limiting step of the palmitoyl-CoA synthetic pathway. This strongly suggests that RV infectivity is mediated through fatty acid metabolism [49] or selected fatty acids.

Altogether, these data imply a possible antiviral mechanism for t-A1 and t-A3 through modulation of the cannabinoid receptors and subsequent alteration of fatty acid metabolism in the host cell. More studies are required to confirm and expand our knowledge of the RV reducing properties of t-A1 and t-A3. Thus, these compounds potentially could be used to design and develop more efficacious RV therapeutic agents.

## Abbreviations

RV: Rotavirus
FFU: Focus forming units
PFU: Plaque forming units
WB: Western blots
NSP4: Nonstructural protein 4
hpi: Hours postinfection
DMSO: Dimethyl sulfoxide
FBS: Fetal bovine serum
DMEM: Dulbecco minimal essential medium
t-A1: * trans*-Arachidin-1
t-A3: * trans*-Arachidin-3
t-PA: * trans*-Piceatannol
t-Rev: * trans*-Resveratrol
MOI: Multiplicity of infection
TC: Tissue culture
MSV: Modified Murashige and Skoog's medium.
